# A Possible Involvement of Sialidase in the Cell Response of the Antarctic Fungus *Penicillium griseofulvum* P29 to Oxidative Stress

**DOI:** 10.3390/life15060926

**Published:** 2025-06-08

**Authors:** Radoslav Abrashev, Ekaterina Krumova, Penka Petrova, Rumyana Eneva, Yana Gocheva, Stefan Engibarov, Jeny Miteva-Staleva, Vladislava Dishliyska, Galina Stoyancheva, Boryana Spasova, Vera Kolyovska, Maria Angelova

**Affiliations:** 1 Department of Mycology, The Stephan Angeloff Institute of Microbiology, Bulgarian Academy of Sciences, Academician G. Bonchev 26, 1113 Sofia, Bulgaria or rabrashev@microbio.bas.bg (R.A.); e_krumova@microbio.bas.bg (E.K.); j_m@abv.bg (J.M.-S.); vladydacheva@yahoo.com (V.D.); bkasovska@abv.bg (B.S.); 2 Department of General Microbiology, The Stephan Angeloff Institute of Microbiology, Bulgarian Academy of Sciences, Academician G. Bonchev 26, 1113 Sofia, Bulgaria; pepipetrova@yahoo.com (P.P.); rum_eneva@abv.bg (R.E.); yanagocheva@microbio.bas.bg (Y.G.); stefan_engibarov@abv.bg (S.E.); galinadinkova@abv.bg (G.S.); 3 Institute of Experimental Morphology, Pathology and Anthropology with Museum, Bulgarian Academy of Sciences, Academician G. Bonchev 25, 1113 Sofia, Bulgaria; verakol@abv.bg

**Keywords:** fungi, Antarctica, sialidase, cold stress, oxidative stress biomarkers, antioxidant enzymes

## Abstract

Sialidases/neuraminidases remove terminal sialic acid residues from glycoproteins, glycolipids, and oligosaccharides. Our previous research has revealed the distribution of sialidase in non-clinical fungal isolates from different ecological niches, including Antarctica. Fungi adapted to extremely low temperatures possess defense mechanisms necessary for their survival such as the response against oxidative stress. The relationship between oxidative stress and sialidase synthesis has been studied extremely sparsely. The aim of the present study was to investigate the involvement of sialidase in the cell response of the Antarctic strain *P. griseofulvum* P29 against oxidative stress induced by long- and short-term exposure to low temperatures. The changes in growth temperatures for 120 h (long-term stress) affected biomass accumulation, glucose consumption, sialidase synthesis, and the activity of the antioxidant enzymes superoxide dismutase (SOD) and catalase (CAT). The short-term temperature downshift (6 h) caused oxidative stress, evidenced by changes in the levels of biomarkers, including lipid peroxidation, oxidatively damaged proteins, and the accumulation of reserve carbohydrates. Simultaneously, a sharp increase in SOD and CAT activity was found, which coincided with a significant increase in sialidase activity. This study marks the first demonstration of increased sialidase activity in filamentous fungi isolated from extreme cold environments as a response to oxidative stress.

## 1. Introduction

The enzymes belonging to the sialidase family, also referred to as neuraminidases or N-acetylneuraminic acid hydrolases (EC 3.2.1.18), catalyze the hydrolysis of terminal neuraminic acids linked to glycoproteins, glycolipids, polysaccharides, mucopolysaccharides, and oligosaccharides via -α2,3, α2,6, or α2,8 linkages [1,2]. They are extensively prevalent in nature and are present in diverse bacteria, viruses, protozoa, and animals [3,4].

However, the production of sialidase by fungi has been very rarely reported. The first report of the presence of sialidase in fungi was made by Japanese scientists who identified the enzyme in the species *Sporotrichium schenckii* and *Penicillium griseofulvum* (formerly known as *Penicillium urticae*) [5]. Sialidase production has been identified in some fungal pathogens such as *Candida albicans* [6], *Aspergillus fumigatus* [7], *Penicillium chrysogenum* [8,9], *Aspergillus terreus*, and *Trichophyton rubrum* [10]. In our previous study, 113 fungal strains isolated from different non-clinical sources were screened. Among them, 77 strains belonging to the phyla *Ascomycota* and *Zygomycota* showed sialidase synthesis [11]. For the first time, the synthesis of sialidase has been reported for fungi isolated from extreme cold conditions, such as those found in Antarctica and Alaska. The most effective producer, *Penicillium griseofulvum*, was selected for the structural and functional characterization of the purified enzyme [12].

In the context of the established cold-adapted isolates producing sialidase, the significance of this enzyme in facilitating fungal survival in extremely cold habitats is a subject of interest. It is well-documented that low temperatures contribute to an increased generation of reactive oxygen species (ROS), which include free radicals such as the superoxide anion (^•^O_2_^−^) and hydroxyl radical (•OH), along with non-radical compounds like hydrogen peroxide (H_2_O_2_) and singlet oxygen (^1^O_2_) [13,14]. This higher level of ROS exceeds the antioxidant defense capacity of the cell and induces the realization of oxidative stress (OS) [15]. Because of OS, damage to all major cellular molecules such as DNA, proteins, and lipids was found. In addition to this, modifications of cell membranes, as well as morphological, physiological, and biochemical changes, are also observed [16].

Microorganisms adapted to extremely low temperatures possess protective mechanisms necessary for their survival [17]. Adaptation to permanently cold habitats is associated with the synthesis of enzymes with high activity at low temperatures [18,19], a change in the lipid composition of cell membranes to preserve their functions, the synthesis of RNA chaperones that suppress the formation of secondary RNA structures, and the synthesis of “cold shock” proteins [20]. Under conditions of low-temperature stress, the synthesis of many proteins is suppressed, and the production of the so-called “cold-shock” proteins is activated, which is probably responsible for the adaptation to low temperatures.

The relationship between oxidative stress and sialidase synthesis has been studied extremely poorly. A limited number of studies have been published, with the study of cell cultures and pathogenic microorganisms reporting the involvement of this enzyme in the cellular stress response [21,22]. Cirillo et al. [23] found accelerated expression of sialidase in HELA cells exposed to hypoxic conditions. The authors support the hypothesis that endosomes in eukaryotic cells are a reservoir of the enzyme, which is ready, although inactive, to be moved to the outer cell membrane when its activity is required, for example, in response to stress conditions. It has been shown that the survival probability and virulence of a mutant *Porphyromonas gingivalis* with sialidase deficiency are reduced under stress conditions (temperature, presence of oxidants, etc.) in the environment compared to those of the parental strain [24]. Furthermore, pathogenic bacteria undergo cellular and physiological changes to survive in the stressful conditions of the microenvironment, including the regulation of the transcription of genes responsible for the synthesis of sialidase. There are reports that sialic acid can neutralize free oxyradicals [25,26] induced by the inflammatory response [27].

Studies on fungi have been documented in isolated reports of pathogenic strains. Warwas et al. [28] reported that sialidase is involved in the cellular response to oxidative/osmotic stress in the pathogenic strain *Aspergillus fumigatus*. The study conducted by Nesbitt et al. [29] highlighted the significant function of *Aspergillus fumigatus* sialidase (Kdnase) in preserving the stability of hyphal cell walls when exposed to oxidative stress conditions. However, there is no evidence of this association in non-clinical isolates or isolates from extremely harsh conditions.

The objective of the present study was to investigate the impact of the sialidase of the Antarctic strain *P. griseofulvum* P29 on the growth and development of the fungus under long- and short-term exposure to low temperatures. To this end, this study examined stress biomarkers and the cell response to cold stress. Furthermore, the activity of the antioxidant defense enzyme was determined.

## 2. Materials and Methods

### 2.1. Fungal Strain and Culture Conditions

The fungal strain *P. griseofulvum* P29 (with an optimal growth temperature of 25 °C), isolated from a soil sample taken from Terra Nova Bay, Antarctica, was used for the experiments. The strain was deposited in the National Bank for Industrial Microorganisms and Cell Cultures, Bulgaria (NBIMCC 9106). Long-term preservation was carried out in the Microbank system (Prolab Diagnostics, Richmond Hill, ON, Canada) consisting of sterile vials that contain 25 porous, colored beads and a cryopreservative fluid at −80 °C. Before use, the conidiospores were grown on Beer agar at 28 °C for 7 days.

For long-term exposure to low temperatures, cultivation was performed in the 3 L bioreactor ABR-09 developed and constructed by the former Central Laboratory for Bioinstrumentation and Automatisation (CLBA) of the Bulgarian Academy of Sciences. The bioreactor was equipped with temperature, pH, and dissolved oxygen (DO) automatic monitoring equipment and a control system. Cultivation was carried out on the Wh medium [11] in two stages. For the first stage, 74 mL of Wh medium was inoculated with 6 mL of the spore suspension at a concentration of 2 × 10^8^ spores/mL in 500 mL Erlenmeyer flasks at 25 °C for 24 h on a rotary shaker (220 r.p.m.). For the second stage, 200 mL of the first-stage culture was brought into the 3 L bioreactor containing 1800 mL of the Wh medium. The cultures were grown at 15, 20, and 25 °C with a stirrer speed of 400 rpm and an airflow of 0.5 v.v.m for 120 h. Samples were collected at 12 h intervals to assess biomass, glucose uptake, and the activity of the enzymes sialidase, SOD, and catalase.

Short-term experiments were carried out in the 3 L bioreactor at the optimal temperature (25 °C) for 36 h (early exponential growth phase). Subsequently, a temperature downshift was applied to 10 and 15 °C. After 6 h of incubation under cold stress conditions, the temperature was upshifted to the optimal value. The control variants were grown at an optimal temperature throughout the whole period. The levels of the stress biomarkers (ROS content, lipid peroxidation, oxidatively damaged proteins, and the accumulation of reserve carbohydrates), as well as the activity of the enzymes sialidase, SOD, and CAT, are determined at two-hour intervals.

### 2.2. Cell-Free Extract Preparation

The cell-free extract was prepared as previously described [30]. All of the steps were performed at 0–4 °C. Briefly, mycelium biomass was harvested by filtration, washed in distilled H_2_O and then in cold 50 mM potassium buffer (pH 7.8), and resuspended in the same buffer. The cell suspension was disrupted by the homogenizer model ULTRA Turrax T25 IKA WERK. The temperature during treatment was maintained at 4–6 °C by chilling in an ice–salt bath and by filtration through a Whatman filter No.4 (Clifton, NJ, USA). Cell-free extracts were centrifuged at 13,000× *g* for 20 min at 4 °C.

### 2.3. Measurement of Stress Biomarkers

The method of superoxide dismutase-inhibited reduction of cytochrome c by Hassan and Fridovich [31], was used to measure the ^•^O_2_^−^ production rate with some modifications described by Kostadinova et al. [32]. For the measurement of hydrogen peroxide production, the method by Pick and Mizel [33] was used.

The glycogen and trehalose content was determined following the procedure by Becker [34] and Vandercammen et al. [35], as modified by Parrou et al. [36]. Soluble reducing sugars were determined by the Somogy–Nelson method [37]. Protein oxidative damage was measured spectrophotometrically as the protein carbonyl content using the 2,4-dinitrophenylhydrazine (DNPH) binding assay [38], as slightly modified by Adachi and Ishii [39].

The carbonyl content was calculated using a molar extinction coefficient of 21 mM^−1^ cm^−1^ as the nanomoles of DNPH incorporated (protein carbonyls) per mg of protein. The protein content was estimated by the Lowry procedure [40] using a solution of bovine serum albumin as the standard. The level of lipid peroxidation was measured by a lipid peroxidation assay kit (Sigma–Aldrich).

Lipid degradation caused by oxidative damage is measured by reacting malondialdehyde (MDA) with thiobarbituric acid (TBA) to produce a colorimetric product proportional to the available MDA. The measurement is performed spectrophotometrically at 532 nm.

### 2.4. Enzyme Activity Determination

The sialidase activity was measured quantitatively by colorimetric determination of free sialic acid by the thiobarbituric acid method of Uchida et al. [41]. One unit of enzyme activity was defined as the amount that releases 1 μg of N-acetyl-neuraminic acid for 1 min at 37 °C, using the glucomacropeptide [42] as a substrate. SOD activity was measured by the nitro-blue tetrazolium (NBT) reduction method of Beauchamp and Fridovich [43]. One unit of SOD activity was defined as the amount of enzyme required for 50% inhibition of NBT reduction (A_560_) and expressed as units per mg protein [U/mg protein]. Catalase activity was determined by monitoring the decomposition of 18 mM H_2_O_2_ at 240 nm [44]. One unit of activity is the amount that decomposes 1 μmol of H_2_O_2_ per minute per mg protein at 25 °C and pH 7.0. Specific activity is given as U/mg protein.

### 2.5. Other Methods

Dry weight determination was performed on samples of mycelia harvested throughout the culture period. The culture fluid was filtered through a Whatman no. 4 filter. The separated mycelia were washed twice with distilled water and dried to a constant weight at 105 °C.

### 2.6. Statistical Evaluation of the Results

The results obtained in this investigation were evaluated from at least three repeated experiments using three parallel runs and reported values representing the mean. The error bars indicate the standard deviation (SD) of the means of triplicate experiments. The data were analyzed using one-way analysis of variance (ANOVA), followed by Tukey’s test.

## 3. Results

### 3.1. Cell Response to Long-Term Cold Stress

#### 3.1.1. Effect of Temperature on Growth and Glucose Consumption

**Figure 1:**
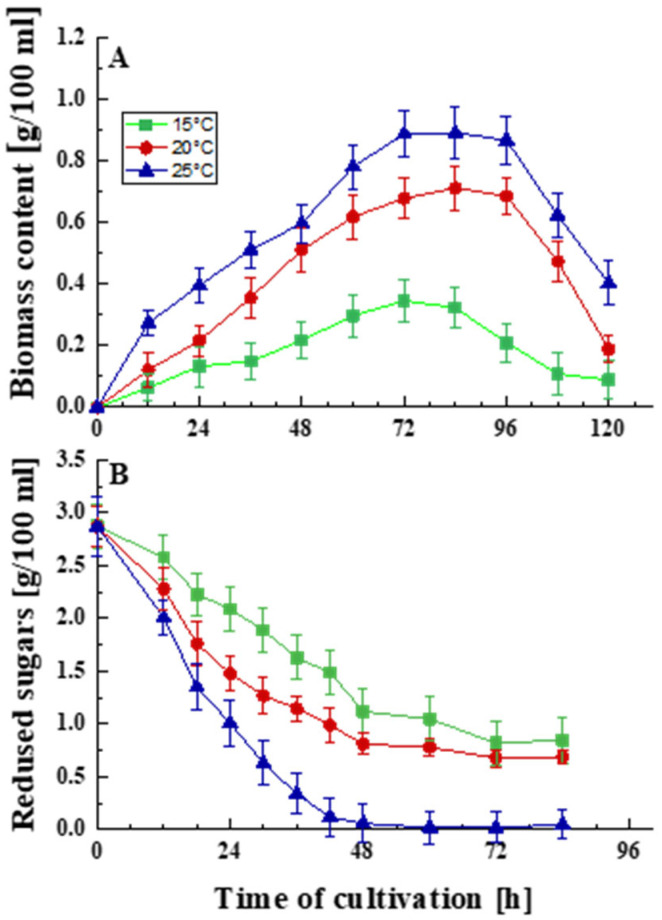
Effect of temperature on the growth (**A**) and glucose uptake (**B**) of *P. griseofulvum* P29. Values are the means of three replicates; error bars represent the standard deviation. Temperatures below the optimal caused a statistically significant effect (Tukey’s test *p* < 0.05) on the growth and glucose uptake (*p* ≤ 0.05).

The cultivation of *P. griseofulvum* P29 for 120 h in 3 L bioreactors at the optimal temperature as well as at temperatures below the optimum characterized the cellular response to prolonged cold stress (Figure 1). The strain demonstrated growth over a range of temperatures consistent with the properties of psychrotolerant fungi (Figure 1A).

The optimal temperature for the development of the strain is 25 °C, but it can develop relatively well at temperatures below the optimum, namely 15 and 20 °C, respectively. Growth curves typical of filamentous fungi were outlined in all experiments. The maximum amount of biomass was accumulated at 72–84 h. Decreasing the temperature led to a shortening of the exponential and stationary phases and a faster entry into the death phase.

Following these results, differences in glucose uptake were also established (Figure 1B). Temperature levels other than the optimal caused physiological changes, as was shown by a reduction in glucose intake. The rapid and abundant growth of the model strain coincided with accelerated glucose consumption. Decreasing the cultivation temperature by 5 and 10 °C inhibited the process. This is probably one of the mechanisms of adaptation to temperature stress.

#### 3.1.2. Effect of Temperature on Antioxidant Enzyme Defense and Sialidase Activity

**Figure 2:**
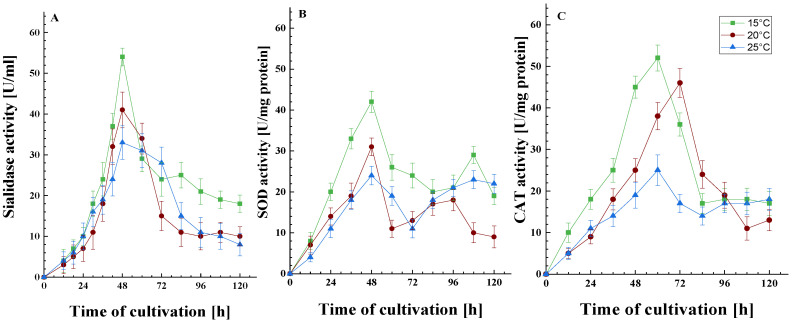
The activity of the sialidase (**A**), SOD (**B**), and CAT (**C**) enzymes as a function of growth temperature. Values are the means of three replicates; error bars represent the standard deviation. Growth temperatures cause a statistically significant effect on the enzyme activities (Tukey’s test *p* ≤ 0.05).

Subsequent experiments provided data on antioxidant enzyme defense (SOD and CAT) and sialidase activity following sub-optimal temperature exposure (Figure 2). As can be seen from Figure 2A, the maximum sialidase activity in the psychrotolerant strain *P. griseofulvum* P29 was not observed at the optimal growth temperature. Cultivation at 15 and 20 °C resulted in a significantly higher level of the enzyme. While the maximum activity at 20 °C was approximately 24% higher than that at the optimal temperature of 25 °C, a 64% increase in activity was observed at 15 °C compared to the control. In other words, the synthesis of sialidase was found to be temperature-dependent.

The data on the dynamics of the synthesis of SOD and CAT are of interest. SOD exhibits two peaks in activity: the first occurred at 48 h during the exponential growth phase, while the second was observed after 84 h, corresponding to the stationary growth phase (Figure 2B). It can be posited that the formation of spores in the bioreactor towards the end of the cultivation period triggers enhanced respiration and, as a result, bolsters the antioxidant enzyme defense mechanisms. This spore development is likely supported by the influx of carbon-containing compounds released from cell lysis. Conversely, CAT exhibited a different activity pattern, with a single peak noted at the end of the exponential phase, followed by a maintenance of activity for 30 to 36 h (Figure 2C). Both antioxidant enzymes exhibited increased activity when subjected to temperatures below their optimal range. This response is likely a crucial aspect of the cellular adaptation to low-temperature stress during the early hours of cultivation, thereby promoting the strain’s growth under these specific conditions.

It is important to note that the level of all three enzymes was affected by the degree of temperature stress, where the higher the degree of stress, i.e., the lower the temperature (15 or 20 °C), the more clearly their involvement in the cellular response was outlined. As is known, the activity of SOD and CAT is affected by the level of free radicals. Consequently, it can be hypothesized that the activity of sialidase will also change following the increase in the amount of ROS.

### 3.2. Oxidative Stress Induction in P. griseofulvum P29 During Short-Term Cold Exposure

The influence of low temperatures was analyzed in the context of short-term stress conditions. A six-hour exposure period was chosen, as preliminary findings suggested that this length of time was sufficient to create a noticeable distinction between control cultures and those subjected to stress.

#### 3.2.1. Biomarkers of Oxidative Stress

- ROS Generation

To elucidate the relationship between low-temperature exposure and oxidative stress, the alterations in the level of generated ROS in cells of the exponential growth phase of *P. griseofulvum* P29 were examined. Table 1 illustrates the changes in the levels of ^•^O_2_^−^ and H_2_O_2_ following a drastic decrease in temperature.

As evident in the findings, the generation of ROS is also observed when the cells are cultivated at the optimal temperature. This phenomenon has been observed in all aerobic cells, including fungal cells. However, a notable increase in the levels of ^•^O_2_^−^ and H_2_O_2_, reaching approximately 30% higher than the control level, is observed when the temperature is reduced from 25 to 15 °C. The accelerated rate of ROS generation is particularly pronounced following a two-hour exposure period to a temperature of 6 °C. The results indicate up to 3.3- and 2.1-fold increases in the levels of ^•^O_2_^−^ and H_2_O_2_, respectively, compared to the control variant.

- Effect on Protein Oxidation

**Figure 3:**
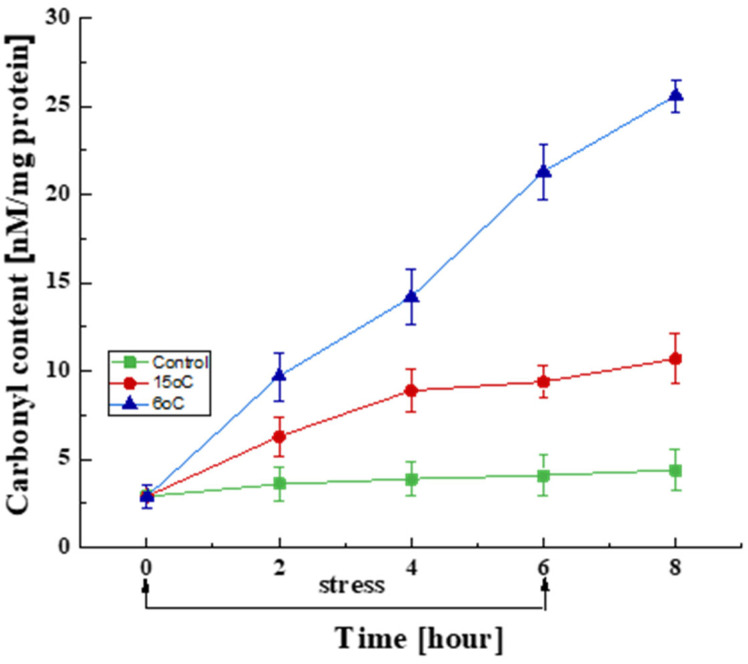
Changes in the carbonyl content in cells exposed to the optimal temperature (■) and transient downshift to 15 °C (●) and 6 °C (▲). Values are the means of three replicates; error bars represent the standard deviation. Decreased temperatures cause a statistically significant increase in oxidatively damaged protein content (Tukey’s test *p* < 0.05).

Changes in the amount of oxidatively damaged proteins during the 6 h stress period and in the recovery phase at the optimal development temperature were studied (Figure 3). A certain amount of carbonyl groups was found in the control variant even at the optimal temperature, which is associated with the generation of ROS under normoxic conditions. In this case, the content of carbonyl groups did not demonstrate significant changes over a period of 6 h. The variants with reduced temperatures (15 and 6 °C), however, already exhibited a significant increase in this biomarker in the first 2 h, which continued until the end of the stress conditions. This trend persisted even after the culture was returned to the optimal temperature. The temperature decreases to 15 and 6 °C led to 3.2- and 7.3-fold increases in the amount of carbonyl groups, respectively, compared to the control variant. It should be noted that the 2 h exposure period at 6 °C caused a two-fold increase in oxidative damage compared to that at 15 °C.

- Effect on MDA Content

**Figure 4:**
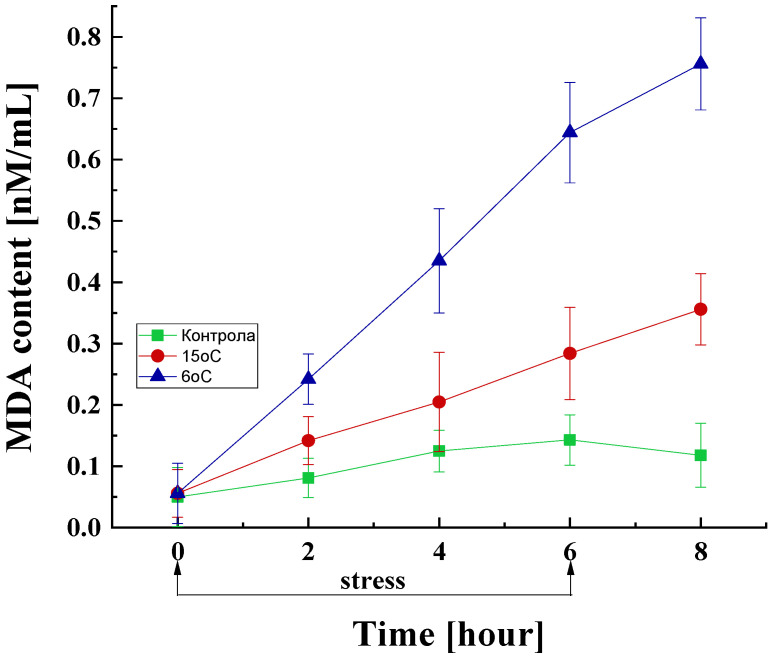
MDA content in the fungal cell exposed to the optimal temperature and short-term downshift to 6 or 15 °C. Values are the means of three replicates; error bars represent the standard deviation. Decreased temperatures cause a statistically significant increase in MDA content (Tukey’s test *p* < 0.05).

MDA, a by-product of lipid peroxidation, was measured to establish the role of lipid peroxidation in the cold-stress response. The results showed that the temperature downshift from the optimal to 15 or 6 °C causes substantial changes in the MDA content in the mycelium of *P. griseofulvum* P29 (Figure 4).

The control variant also showed an inevitable increase in the amount of MDA even under the optimal temperature conditions. In the cultures subjected to low-temperature stress, a significant increase in the MDA content was observed compared to the control, which is proportional to the duration of cultivation under stress conditions and the degree of stress. Specifically, within the initial 2 h of stress exposure, MDA levels in these cultures escalate by approximately 1.8- and 3-fold at 15 and 6 °C, respectively, compared to the control group. After 4 and 6 h of stress exposure, a substantial surge in lipid peroxidation levels was observed, with cultures exposed to 6 °C demonstrating levels 3.5- to 4.5-fold higher compared to the control at equivalent time points. Notably, even after the cessation of stress, a similar increasing trend was observed, with the MDA content reaching approximately 6.5-fold higher levels compared to the control.

- Changes in Reserve Carbohydrates

**Figure 5:**
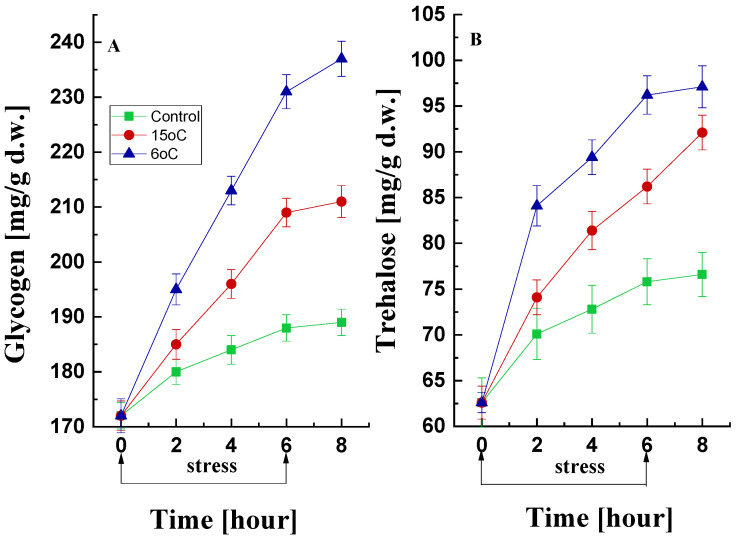
Changes in glycogen (**A**) and trehalose (**B**) content during cold stress and recovery in *P. griseofulvum* P29 upon the temperature downshift from the optimal to 15 °C or 6 °C. Values are the means of three replicates; error bars represent the standard deviation. The effect on reserve carbohydrates was significant for the temperature treatment (Tukey’s test *p* > 0.05).

The accumulation of trehalose and glycogen in microbial cells under adverse growth conditions can serve as indicators of stress. The effect of low-temperature treatment on glycogen and trehalose production by the psychrotolerant strain *P. griseofulvum* P29 was studied (Figure 5). In the control variant, a tendency to maintain glycogen content within a narrow range (172–188 mg/g d.w.) was found (Figure 5A). However, the temperature downshift to 15 °C or 6 °C caused a sharp increase in the first 2 h (from 172 to 185 and 195 mg/g d.w., respectively). This increase continued exponentially until the 6th hour. Following the cessation of stress, a trend of preservation of the amount of glycogen was observed (between 6 and 8 h).

Similar changes were observed in the trehalose data (Figure 5B). Mycelia cultured at the optimal temperature also accumulated trehalose, with a 20% increase recorded over 6 h. Much more pronounced was the increase in the amount of trehalose under low-temperature stress conditions. Exponential accumulation was detected within the initial 2 h of exposure either to 15 °C or 6 °C. This accumulation reached approximately 40% and 53%, respectively, by the conclusion of the 6 h period, in comparison with the baseline value.

#### 3.2.2. Changes in the Activity of Antioxidant Enzymes

**Figure 6:**
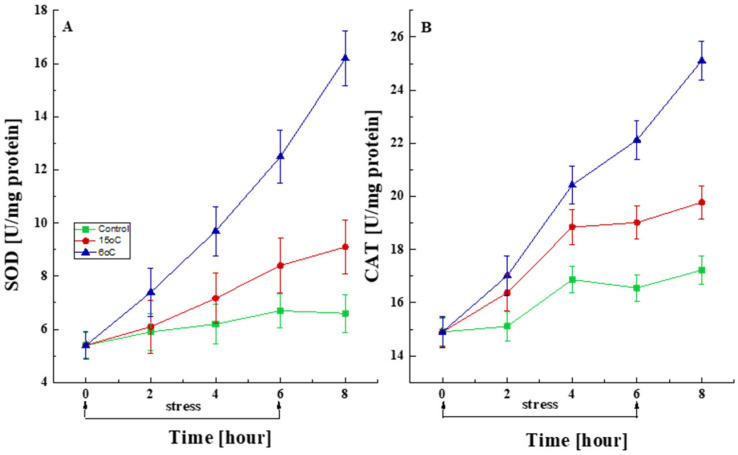
Changes in the activity levels of SOD (**A**) and CAT (**B**) after short-term treatment with the optimal temperature and at the corresponding stress temperatures (6 °C or 15 °C). Values are the means of three replicates; error bars represent the standard deviation. The temperature caused a statistically significant effect on SOD and CAT activity (Tukey, *p* > 0.05).

To assess the changes in antioxidant protection, the activity of the antioxidant enzymes SOD and CAT was measured in cells subjected to transient low-temperature stress (15 °C or 6 °C) for 6 h (Figure 6). A statistically significant increase in SOD activity was observed in response to the temperature downshift when compared with control cultures (Figure 6A). Furthermore, *P. griseofulvum* P29 exhibited a rapid and significant response to temperature change as early as the first 2 h. The increase in SOD activity persisted until the 6th hour and even after the cessation of exposure.

Short-term exposure to low temperatures also resulted in statistically increased CAT activity compared to control cultures. As seen in Figure 6B, the accelerated synthesis of CAT began immediately after the stress exposure and continued with equal intensity after its cessation.

#### 3.2.3. Effect of Temperature Downshift on Sialidase Activity

**Figure 7:**
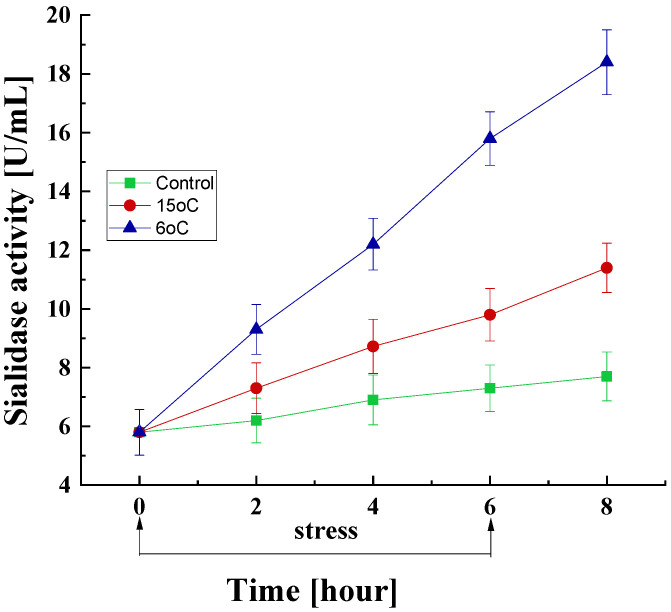
Sialidase activity response of *P. griseofulvum* P29 to a transient decrease in the growth temperature from the optimal to 15 °C or 6 °C. Values are the means of three replicates; error bars represent the standard deviation. The temperature downshift resulted in a statistically significant increase in enzyme activity.

It is of interest to study the changes in sialidase activity under short-term low-temperature stress conditions. In the mycelia of *P. griseofulvum* P29 cultured at the optimal temperature for six hours, a slight change in sialidase activity was observed, from 5.8 to 7.3 U/mL (approximately 25%) (Figure 7).

Conversely, there was a marked increase in sialidase levels within the first 2 h after a sharp drop in temperature to 15 °C or 6 °C. This increase was found to be significantly more pronounced at 6 °C compared with that at 15 °C. Following a 6 h exposure period to 15 °C, sialidase activity exhibited a 1.7-fold increase compared to the baseline level (9.8 vs. 5.8 units/mL) and a 1.34-fold increase compared to the control group at the same time point (9.8 vs. 7.3 units/mL). At 6 °C exposure, these values were 2.7- and 2.1-fold, respectively. The increase in sialidase activity was also observed at the eighth hour, i.e., after the cessation of low-temperature stress.

## 4. Discussion

The present study provides information on the cellular response of the Antarctic strain *P. griseofulvum* P29, a sialidase producer, to low-temperature stress. Decreases in temperature are considered to be abiotic stresses that have been demonstrated to induce the generation of ROS in prokaryotic and eukaryotic cells. In this context, a reduction in metabolic activity resulted in diminished ATP consumption, the accumulation of electrons at specific locations in the respiratory chain, and accelerated generation of ROS (^•^O_2_^−^, H_2_O_2_, and OH•) [45,46]. These ROS have been observed to damage growth and all cellular components, including DNA, lipids, and proteins.

Information on the cellular response of microorganisms isolated from extremely cold habitats to low-temperature stress is very scarce [47,48]. Even less is known about filamentous fungi isolated from Antarctica. Our previous studies have demonstrated the induction of oxidative stress in Antarctic fungi as a result of exposure to low temperatures [30,49,50,51,52,53,54]. The present study centered on a psychrotolerant strain, *P. griseofulvum* P29, obtained from Antarctic soil. The experiments were designed to obtain information by exposure to both long- and short-term cold stress.

### 4.1. Cell Response of Antarctic P. griseofulvum P29 to Long-Term Cold Stress

The results from long-term cold stress demonstrated that the model strain grew well in the wide range of temperatures showing an optimum at 25 °C. According to the definition by Morita [55], this strain belongs to psychrotrophic (psychrotolerant) microorganisms. A substantial body of the literature has indicated that psychrotolerant fungi constitute the predominant component of the Antarctic mycobiota [56,57,58,59]. Their occurrence in cold environments may be due to solar radiation, which induces seasonal and daily increases in soil temperature, thus supporting the development of cold-adapted microorganisms instead of strictly psychrophilic ones [60]. Usually, the ratio of psychrotolerant organisms to psychrophilic ones is found to be 80:20 [61,62]. Mesophilic fungi have also been discovered in Antarctica, where they exist as viable propagules but can only reproduce under infrequent favorable climatic conditions [58,63,64]. In general, the present results regarding the effect of temperature on the growth of the Antarctic strain *P. griseofulvum* P29 showed that it is psychrotolerant, indicating a high adaptation to cold ecological niches.

The temperature dependence of growth was also observed for glucose uptake. The findings indicated that the model strain exhibited the highest glucose utilization rate during the initial 48 h of cultivation at the optimal temperature of 25 °C, with a decline in utilization observed at temperatures below this optimum. The role of glucose consumption in the cell response to cold stress has been reported for the Antarctic psychrotolerant strain *Mrakia blollopis*, which exhibited distinct glucose consumption characteristics under low-temperature conditions compared to the optimal one [65]. The combined effects of temperature and glucose also influence the development of cold-adapted *Fusarium sporotrichioides* [66] and *Pseudogymnoascus pannorum* [67]. Using proteomics approaches, Abu Bakar [20] found that the proteins linked to the metabolic activity of general carbon metabolism were reduced as a result of exposure to low-temperature stress. A significant down-regulation of the proteins, such as malate synthase, malate dehydrogenase, acetyl-coenzyme A synthetase, and glyceraldehyde-3-phosphate dehydrogenase, has been determined.

Prolonged exposure to a temperature below the optimal coincided with a significant increase in the activity of antioxidant enzymes, specifically SOD and CAT (Figure 2B,C). It is important to note that these changes were dependent on the stress level. Fungi have been observed to establish antioxidant defense mechanisms that neutralize ROS and protect cells from oxidative stress damage. Thus, modification in the levels of antioxidants may serve as a useful marker for assessing oxidative stress levels and the degree of stress resistance. The present results demonstrated that the psychrotolerant strain *P. griseofulvum* P29 exhibited a heightened antioxidant response to a growth temperature of 15 °C compared to 20 °C. At the same time, data were obtained that suggest the involvement of sialidase in the cellular response to temperature stress. Changes in the activity of this enzyme at sub-optimal temperatures are similar to those reported for SOD and CAT.

However, there is a scarcity of research investigating the relationship between sialidase levels and the level of abiotic stress. There is much less data on the subject of fungi. Bateman et al. [68] published data on increased sialidase activity in certain bacteria (*Pseudomonas aeruginosa*, *Clostridium perfringens*, and *Vibrio cholerae*) under conditions of biotic stress (sepsis). Other researchers working in the field of sepsis [69] have also reported similar findings. As demonstrated in the study by Xu et al. [24], the survival, virulence, and biofilm formation of a sialidase-deficient mutant of *P. gingivalis* are reduced under conditions of stress, including high temperatures, in comparison to the wild strain. However, there are no reports of experiments under cold stress conditions. Our results are the first in this direction.

### 4.2. Transient Temperature Downshift Induces Oxidative Stress in Antarctic Strain P. griseofulvum P29

#### 4.2.1. Changes in Oxidative Stress Biomarkers

The outcomes of long-term cold stress were confirmed by those attained following six hours of exposure to low temperatures (short-term stress). As can be seen from Table 1, the present study provided information regarding the accelerated generation of ROS in the cells of the strain *P. griseofulvum* P29 under low-temperature conditions. It is known that the unstressed fungal cells are capable of producing ^•^O_2_^−^ and H_2_O_2_, likely due to a single-electron reduction of 2% of the consumed oxygen [70,71]. Our direct experiment demonstrated a considerable rise in ^•^O_2_^−^ after a temperature downshift from the optimal temperature to 15 °C, or 6 °C in a dose-dependent manner. A similar but less pronounced tendency was reported for H_2_O_2_ levels. Therefore, cold stress presumably imposes an oxidative load of which ^•^O_2_^−^ is a substantial component. To the best of our knowledge, a comparable direct analysis of the ROS content in fungal cells has rarely been documented. In most cases, it concerns abiotic stress. There is a lack of data to determine the change in the level of ROS when exposed to temperature. Da Silva et al. [72] detected ROS generation in *Fusarium oxysporum* and *F. silani* by fluorescence microscopy after the treatment with a peptide fraction from *Capsicum chinense* Jack. A similar method has been used to measure ROS levels in *Aspergillus fumigatus* in response to the application of antifungal agents [73]. Exposure to 4 mM H_2_O_2_ for 60 min resulted in enhanced production of ^•^O_2_^−^ in *Saccharomyces cerevisiae* [74]. The research by Papapostolou and Georgiou [75] showed increased levels of superoxide radicals in the phytopathogenic fungi *Rhizoctonia solani, Sclerotinia sclerotiorum*, *Sclerotium rolfsii*, and *Sclerotinia minor* during the process of cell differentiation. As demonstrated in the relevant literature, changes in the level of ROS have been shown to occur in response to various stimuli, including exposure to heavy metals [76,77], elevated temperatures [78], cold stress [52], etc.

Consequently, an elevated concentration of ROS after a drastic temperature downshift resulted in the realization of oxidative stress in *P. griseofulvum* P29 cells, as evidenced by changes in the levels of stress biomarkers. A very rapid increase in the amount of oxidatively damaged proteins was found when the temperature was sharply reduced from the optimal to 15 °C or 6 °C compared to the control variants. As is known, one of the ways in which intracellular proteins are modified by ROS is the formation of additional carbonyl groups [79]. Radicals oxidize specific amino acid residues (Arg, His, Lys, Pro, Thr, and Trp) in the protein backbone, resulting in the formation of free carbonyl groups. This, in turn, leads to the inhibition of their physiological activity and increases their susceptibility to proteolytic attack.

The measurement of carbonyl groups has been demonstrated to be a valid method for assessing the impact of stress on cells [80]. It may provide correct data to assess the level of stress. In accordance with the findings of numerous studies [20,52,53,54,81], the present study demonstrates the relationship between higher levels of ROS and increased amounts of oxidatively damaged proteins in the Antarctic fungal strain. A comparable correlation has been identified in other organisms, such as bacteria [82,83], plants [84,85], tumor cells [86], etc. As demonstrated by Ouellet [87] and Suzuki and Mittler [88], changes in plant cellular proteins have been observed as part of the process of adaptation to extremely low temperatures. Analogous cellular responses have been observed in hyperoxal cultures of *Phanerochaete chrysosporium* [89] upon exposure to heavy metals, exogenous addition of redox-active compounds, and changes in temperature [90]. Li et al. [91] have demonstrated that temperature-induced protein carbonylation primarily affects respiratory enzymes and those located in mitochondria, as the respiratory chain ROS are the main source of oxidative damage observed during periods of stress. Furthermore, the proteomic analyses identified a positive correlation between the ROS level and both the composition and sequences of amino acids [83,92].

The present results showed also that the changes in the amount of carbonylated proteins depend on the degree of stress. A notable increase in the response is observed when the temperature is reduced to 6 °C, as compared to the response at 15 °C. Differential expression of cold acclimation proteins (Caps) and cold shock proteins (Csps) may be the underlying reason for the varied cellular responses to both temperatures [93,94,95]. Numerous Csps involved in multiple cellular activities (transcription, translation, protein folding, and the regulation of membrane fluidity) may function as Caps that are constitutively expressed at low temperatures rather than transiently.

Low-temperature stress induces significant changes in the amount of reserve carbohydrates in prokaryotic and eukaryotic cells, which can be employed as a biomarker for stress evaluation [96]. Published information suggests that microbial cells accumulate glycogen and trehalose under unfavorable conditions [97]. These carbohydrates are important protective substances in vegetative fungal cells and spores. Increased synthesis is part of the cellular response to potentially lethal factors such as temperature, osmotic pressure, heavy metals, antifungal agents, and other stressors [98,99]. The findings of the present study demonstrate that trehalose and glycogen are indispensable for the development of the *P. griseofulvum* P29 strain isolated from an extreme cold habitat. When cultured under low-temperature stress conditions, a reduction in glucose uptake was accompanied by a significant increase in trehalose and glycogen content. This finding aligns with previous observations made by other researchers who have reported a similar correlation in response to diverse types of abiotic stress [36,100,101].

In the present experiments, the amount of the two reserve carbohydrates began to increase simultaneously and immediately after the onset of stress. This is in contrast to the data reported previously, in which it was demonstrated that glycogen accumulated earlier and faster compared to trehalose [102]. The observed discrepancy indicates the existence of a precise regulatory mechanism during the stress period, which is contingent on various factors, including the type of culture, the nature of the stressor, and the duration of stress [100]. It has been demonstrated that the signaling pathways for the activation of reserve carbohydrate synthesis under different types of stress do not overlap. Furthermore, the level of these two carbohydrates is determined by the balance between their synthesis and degradation within the cell, following changes in carbon metabolism.

The mechanism by which reserve carbohydrates protects the cell from the effects of stress is poorly understood. It has been suggested that trehalose minimizes oxidative damage to proteins and lipids that would otherwise be degraded by the effects of ROS [103,104]. Trehalose slows down the formation of protein aggregates, and its presence in the lipid bilayer is mandatory. Glycogen also plays an important role in the survival of microorganisms including mycelia during starvation and sporulation, when entering the stationary phase of development, and upon exposure to many stresses. It has been suggested that this carbohydrate is involved in the coordinated control of the glycolytic pathway during stress.

Intracellular ROS induce lipid oxidation, commonly termed lipid peroxidation, which is one of the most frequent outcomes of oxidative stress. This process involves a series of oxidative degradation reactions [81]. At the same time, the ROS produced can function as signaling molecules or interact with other biomolecules within the cell, causing various types of damage, particularly to cellular membranes [105]. Such damage may lead to alterations in membrane permeability and fluidity, a reduction in the stability of lipid–protein complexes, and the inactivation of membrane-bound enzymes that rely on lipids.

The increased amount of MDA in the cells of the Antarctic strain *P. griseofulvum* P29 is consistent with several studies on temperature stress, heavy metal stress, etc. [106,107]. MDA is a criterion for the level of lipid peroxidation. Oxidation products overproduced by lipid peroxidation accumulate in the cells and can lead to organelle dysfunction, especially dysfunctional endoplasmic reticulum, mitochondria, lysosomes, and peroxisomes. As a result, more ROS are generated, which increase oxidative damage and accelerate changes in cells. Similar results have been published mostly for plants [108,109]. The treatment of tea plants (*Camellia sinensis*) with temperatures of 4 °C and 1 °C induced MDA accumulation depending on the duration of the stress [110]. Furthermore, the MDA content exhibited a significantly more pronounced increase at 1 °C in comparison with 10 °C. The authors suggested that low temperatures could damage leaf cell membranes through a process of lipid peroxidation. In a related study, Raza et al. [111] reviewed the relationship between cold exposure and an increase in ROS generation and MDA content in various plant species. They emphasized the impact of the upregulation of antioxidant enzyme expression. The process of lipid peroxidation under cold stress has also been proven in bacteria [112,113]. Supporting these results, Naguib et al. [114] demonstrated lipid peroxidation in *Burkholderia cenocepacia* and the role of gene encoding a cytochrome b_561_ membrane protein in cell envelope protection. Similar information was rarely found for fungi, especially for the effect of temperature. ROS-induced oxidative stress has been noted as a reason for the increase in lipid peroxidation in *Fusarium solani* [115], *Glomus intraradices* [116], *Glomus irregulare* [117], etc. The change in lipid compositions of membranes has been characterized as bacterial and fungal strategies during extreme environmental conditions [118,119,120,121].

#### 4.2.2. Antioxidant Enzyme Response to Low Temperature

The antioxidant enzyme defense system is a very important mechanism for the survival of microorganisms, including filamentous fungi, under conditions of abiotic stress. The present study proved a sharply enhanced SOD and CAT activity in the Antarctic strain *P. griseofulvum* P29 after a temperature downshift. The cell response exhibited a dependence on stress level and exposure time. The elevated levels of SOD and CAT have been demonstrated to be associated with the induced resistance of fungi to various stressors [108,122,123]. Their activity neutralizes ^•^O_2_^−^ and H_2_O_2_, both of which tend to accumulate under stress conditions. Additionally, at lower temperatures, the solubility of gases increases significantly, including that of oxygen, which is a highly reactive molecule. The increased level of dissolved oxygen leads to the generation of a greater amount of ROS and hence the activation of SOD and CAT synthesis.

Evidence suggested that a comparable increase in the level of antioxidant enzymes is observed in response to low-temperature exposure for many prokaryotic and eukaryotic organisms [81,124]. Prior research conducted within our laboratory has shown that Antarctic fungi similarly engage antioxidant defense mechanisms in their cellular response to low growth temperatures [31,50,51,53]. The Antarctic strains *P. rubens* III11-2 and *A. fumigatus* 1–9 exhibited enhanced extracellular SOD and CAT activity when cultivated at 15 °C in comparison to 25 °C. Fiedurek et al. [125] reported that cold-adapted *P. cyclopium* 1 demonstrated more pronounced CAT production at 20 °C than at 25 °C and 30 °C. Comparable trends in the levels of the antioxidant enzymes, such as glutathione reductase (GR), ascorbate peroxidase (APX), SOD, and CAT, have been shown by arbuscular mycorrhizal fungi (AMFs) [106].

All these data provide further evidence of the link between low temperatures and oxidative stress. In addition, the temperature stability of antioxidant enzymes in the cell is of particular importance. Enzymes that neutralize ROS under conditions of low-temperature stress have a much lower detoxification capacity when exposed to high temperatures, and vice versa. The presence of temperature-sensitive enzymes in Antarctic isolates is thought to be the reason for their greater tolerance to cold stress.

### 4.3. Role of Sialidase in the Cell Response Against Oxidative Stress Induced by Low Temperatures

The findings in the present study also provide information about a sharp increase in the activity of the enzyme sialidase under conditions of oxidative stress induced by low temperatures. This increase is concomitant with the accelerated synthesis of the antioxidant enzymes SOD and CAT. In the literature, there is no data on the role of sialidase in the development of non-clinical strains of filamentous fungi under stress conditions, including low-temperature stress. The sialidase (Kdnase) in the pathogenic strain *A. fumigatus* has been shown to be critical for growth and development under conditions of hyperosmotic stress [29]. The deletion of *kdnase* gene slowed the growth, altered morphology, and weakened the fungal cell wall.

There are reports on the relationship of sialic acids with biomarkers of oxidative stress and the pathogenesis of some diseases, such as diabetes, cancer, etc. Increased sialidase activity, combined with acute stress (influenza virus), suggests desialylation in human blood as a prerequisite for the molecular mechanism of atherogenesis [126]. A study by Yadav et al. [127] demonstrated an increase in the amount of free sialic acids in the blood plasma of patients with Alzheimer’s disease in parallel with parameters of oxidative stress. According to Xu et al. [24], a sialidase-deficient strain of the species *Porphyromonas gingivalis* is more susceptible to oxidative stress compared to the parent strain. Data suggesting that sialidase dynamically and specifically regulates the expression of polysialic acids in the brain has been reported [128]. A consistent result shown by many studies was the relationship between sialic acids and antioxidant capacity [25,129]. Released sialic acid by the hydrolysis of the glycan chain of all cell types can act as an active ROS scavenger that reduces oxidative stress [25]. For example, sialic acids released from mucins neutralize the formed hydroxyl radicals by breaking the glycosyl bond, and an unknown oxidized product is formed [130]. The large amount of terminal sialic acid residues from mucin in the mucus layer covering the epithelial surfaces of the respiratory and gastrointestinal tracts successfully neutralizes the OH^•^ generated by endogenous environmental sources, such as xenobiotics, pesticides, etc. According to Yida et al. [131], N-acetylneuraminic acid (Neu5Ac), a type of sialic acid, can profoundly reduce the oxidative stress level. It should be noted that Neu5Ac exhibited stability at temperatures below 25 °C [132]. Pawluczyk [133] suggested that the unique structure of sialic acids such as α-ketocarboxylic acids is responsible for the chemical scavenge of H_2_O_2_. Upon examining the latest data, Li et al. [134] concluded that sialic acids can influence signaling pathways related to oxidative stress and the expression of crucial target genes, thereby acting as an antioxidant.

## 5. Conclusions

This study provides important insights into the role of sialidase in the cell response of the Antarctic psychrotolerant fungal strain *P. griseofulvum* P29 to cold stress. Long-term and short-term exposure to low temperatures induces events typical of oxidative stress. When the growth temperature is sharply reduced from the optimum (25 °C) to 15 and 6 °C, an increase in the level of stress biomarkers was observed. The results showed an increase in ROS generation, lipid peroxidation, and oxidatively damaged protein levels. In addition, changes in the amount of reserve carbohydrates and antioxidant enzyme activity were evaluated. At the same time, sialidase synthesis was accelerated. This is the first time that increased sialidase activity as a result of oxidative stress has been demonstrated in a filamentous fungus. The sialidase in the strain *P. griseofulvum* P29 is probably involved in the response to low-temperature stress.

## Figures and Tables

**Table 1:** ROS production in the cytosolic fractions of *P*. *griseofulvum* P29 under conditions of cold stress.

Temperature [°C]	^•^O_2_^−^ (μM/mg d.w./h)	H_2_O_2_ (μM/mg d.w./h)
Optimal	5.43 ± 0.61	11.68 ± 0.88
15	7.03 ± 0.84	15.22 ± 0.83
6	18.04 ± 1.07	24.18 ± 0.71

## Data Availability

Data are contained within the article.

## Abbreviations

The following abbreviations are used in this manuscript:

ROS	Reactive oxygen species
SOD	Superoxide dismutase
CAT	Catalase
OS	Oxidative stress
MDA	Malondialdehyde
^•^O_2_^−^	Superoxide anion radical
•OH	Hydroxyl radical

## References

[1] Giacopuzzi E., Bresciani R., Schauer R., Monti E., Borsani G. (2012). New insights on the sialidase protein family revealed by a phylogenetic analysis in metazoa. PLoS ONE.

[2] Nag S., Mandal A., Joshi A., Jain N., Srivastava R.S., Singh S., Khattri A. (2022). Sialyltransferases and neuraminidases: Potential targets for cancer treatment. Diseases.

[3] Schwerdtfeger S.M., Melzig M.F. (2010). Sialidases in biological systems. Pharmazie.

[4] Aljohani M.A., Sasaki H., Sun X.-L. (2024). Cellular translocation and secretion of sialidases. J. Biol. Chem..

[5] Uchida Y., Tsukada Y., Sugimori T. (1974). Production of microbial neuraminidases induced by colominic acid. Biochim. Biophys. Acta.

[6] Royal G.C., Nandedkar A.K., Sampson C.C., Fagget T. (1984). Neuraminidase production by *Candida albicans*. J. Natl. Med. Assoc..

[7] Warwas M.L., Watson J.N., Bennet A.J., Moore M.M. (2007). Structure and role of sialic acids on the surface of *Aspergillus fumigatus* conidiospores. Glycobiology.

[8] Van Dijk A.A., Cyplenkov N.A., Dekker P.J.T., Efimova Y.M. (2011). Enzymes Other than Milk Clotting Enzymes, e.g. Lipase, Beta-Galactosidase. U.S. Patent.

[9] Telford J.C., Yeung J.H.F., Xu G., Kiefel M.J., Watts A.G., Hader S., Chan J., Bennet A.J., Moore M.M., Taylor G.L. (2011). The *Aspergillus fumigatus* sialidase is a 3-deoxy-d-glycero-d-galacto-2-nonulosonic acid hydrolase (KDNase). Structural and mechanistic insights. J. Biol. Chem..

[10] Nejatie A., Steves E., Gauthier N., Baker J., Nesbitt J., McMahon S.A., Oehler V., Thornton N.J., Noyovitz B., Khazaei K. (2021). Kinetic and structural characterization of sialidases (Kdnases) from Ascomycete fungal pathogens. ACS Chem. Biol..

[11] Abrashev R., Krumova E., Petrova P., Eneva R., Kostadinova N., Miteva-Staleva J., Engibarov S., Stoyancheva G., Gocheva Y., Kolyovska V. (2021). Distribution of a novel enzyme of sialidase family among native filamentous fungi. Fungal Biol..

[12] Dolashki A., Abrashev R., Kaynarov D., Krumova E., Velkova L., Eneva R., Engibarov S., Gocheva Y., Miteva-Staleva J., Dishliyska V. (2023). Structural and functional characterization of cold-active sialidase isolated from Antarctic fungus *Penicillium griseofulvum* P29. Biochem. Biophys. Rep..

[13] Halliwell B. (1996). Antioxidants in human health and disease. Annu. Rev. Nutr..

[14] Rauf A., Khalil A.A., Awadallah S., Khan S.A., Abu-Izneid T., Kamran M., Hemeg H.A., Mubarak M.S., Khalid A., Wilairatana P. (2024). Reactive oxygen species in biological systems: Pathways, associated diseases, and potential inhibitors—A review. Food Sci. Nutr..

[15] Duarte A.W.F., dos Santos J.A., Vianna M.V., Vieira J.M.F., Mallagutti V.H., Inforsato F.J., Wentzel L.C.P., Lario L.D., Rodrigues A., Pagnocca F.C. (2018). Cold-adapted enzymes produced by fungi from terrestrial and marine Antarctic environments. Crit. Rev. Biotechnol..

[16] Juan C.A., Pérez de la Lastra J.M., Plou F.J., Pérez-Lebeña E. (2021). The chemistry of reactive oxygen species (ROS) Revisited: Outlining their role in biological macromolecules (DNA, lipids and proteins) and induced pathologies. Int. J. Mol. Sci..

[17] Jahed K.R., Saini A.K., Sherif S.M. (2023). Coping with the cold: Unveiling cryoprotectants, molecular signaling pathways, and strategies for cold stress resilience. Front. Plant Sci..

[18] Feller G., Gerday C. (2003). Psychrophilic enzymes: Hot topics. in cold adaptation. Nat. Rev. Microbiol..

[19] Fenice M. (2016). The psychrotolerant Antarctic fungus *Lecanicillium muscarium* CCFEE 5003: A powerful producer of cold-tolerant chitinolytic enzymes. Molecules.

[20] Abu Bakar N., Karsani S.A., Alias S.A. (2020). Fungal survival under temperature stress: A proteomic perspective. PeerJ.

[21] Volkhina I.V., Butolin E.G. (2022). Oxidative stress and changes in liver sialoglycoconjugate metabolic parameters in rats with alloxanic diabetes mellitus. Diabetes Mellit..

[22] Wang W., Tan Q., Wang Q., Wang J., Zhang F., Zheng X., Yun J., Zhang W., Zhao F. (2025). Glutathione peroxidase gene regulates substrate development and prevents strain aging in *Volvariella volvacea*. Int. J. Biol. Macromol..

[23] Cirillo F., Ghiroldi A., Fania C., Piccoli M., Torretta E., Tettamanti G., Gelfi C., Anastasia L. (2016). NEU3 sialidase protein interactors in the plasma membrane and in the endosomes. J. Biol. Chem..

[24] Xu X., Tong T., Yang X., Pan Y., Lin L., Li C. (2017). Differences in survival, virulence and biofilm formation between sialidase-deficient and W83 wild-type *Porphyromonas gingivalis* strains under stressful environmental conditions. BMC Microbiol..

[25] Iijima R., Takahashi H., Namme R., Ikegami S., Yamazaki M. (2004). Novel biological function of sialic acid (N-acetylneuraminic acid) as a hydrogen peroxide scavenger. FEBS Lett..

[26] Aruni W., Vanterpool E., Osbourne D., Roy F., Muthiah A., Dou Y., Fletcher H.M. (2011). Sialidase and sialoglycoproteases can modulate virulence in *Porphyromonas gingivalis*. Infect. Immun..

[27] Doostkam A., Malekmakan L., Hosseinpour A., Janfeshan S., Roozbeh J., Masjedi F. (2022). Sialic acid: An attractive biomarker with promising biomedical applications. Asian Biomed. Res. Rev. News.

[28] Warwas M.L., Yeung J.H., Indurugalla D., Mooers A.O., Bennet A.J., Moore M.M. (2010). Cloning and characterization of a sialidase from the filamentous fungus, *Aspergillus fumigatus*. Glycoconj. J..

[29] Nesbitt J.R., Steves E.Y., Schonhofer C.R., Cait A., Manku S.S., Yeung J.H.F., Bennet A.J., McNagny K.M., Choy J.C., Hughes M.R. (2018). The *Aspergillus fumigatus* sialidase (Kdnase) contributes to cell wall integrity and virulence in amphotericin b-treated mice. Front. Microbiol..

[30] Gocheva Y.G., Tosi S., Krumova E.T., Slokoska L.S., Miteva J.G., Vassilev S.V., Angelova M.B. (2009). Temperature downshift induces antioxidant response in fungi isolated from Antarctica. Extremophiles.

[31] Hassan H.M., Fridovich I. (1979). Intracellular production of superoxide radical and of hydrogen peroxide by redox active compounds. Arch. Biochem. Biophys..

[32] Kostadinova N., Krumova E., Stefanova T., Dishliyska V., Angelova M., Lushchak V.I., Stoliar O. (2012). Transient cold shock induces oxidative stress events in Antarctic fungi. Oxidative Stress/Book 3.

[33] Pick E., Mizel D. (1981). Rapid microassays for the measurement of superoxide and hydrogen peroxide production by macrophages in culture using an autoenzyme immunoassay reader. J. Immunol. Methods.

[34] Becker J.U. (1978). A method for glycogen determination in whole yeast cells. Anal. Biochem..

[35] Vandercammen A., François J.M., Torres B.B., Maia J.C., Hers H.G. (1990). Fructose 2,6-bisphosphate and carbohydrate metabolism during the life cycle of the aquatic fungus *Blastocladiella emersonii*. J. Gen. Microbiol..

[36] Parrou J.L., Teste M.A., François J. (1997). Effects of various types of stress on the metabolism of reserve carbohydrates in *Saccharomyces cerevisiae*: Genetic evidence for a stress-induced recycling of glycogen and trehalose. Microbiology.

[37] Somogyi M. (1952). Notes on sugar determination. J. Biol. Chem..

[38] Hart P.J., Balbirnie M.M., Ogihara N.L., Nersissian A.M., Weiss M.S., Valentine J.S., Eisenberg D. (1999). A structure-based mechanism for copper-zinc superoxide dismutase. Biochemistry.

[39] Adachi H., Ishii N. (2000). Effects of tocotrienols on life span and protein carbonylation in *Caenorhabditis elegans*. J. Gerontol. A Biol. Sci. Med. Sci..

[40] Lowry O.H., Rosebrough N.J., Farr A.L., Randall R.J. (1951). Protein measurement with the Folin phenol reagent. J. Biol. Chem..

[41] Uchida Y., Tsukada Y., Sugimori T. (1977). Distribution of neuraminidase in *Arthrobacter* and its purification by affinity chromatography. J. Biochem..

[42] Abrashev I., Velcheva P., Nikolov P., Kourteva J. (1980). Substrate for Colorimetric Determination of Enzyme Activity. Bulgarian Patent.

[43] Beauchamp C., Fridovich I. (1971). Superoxide dismutase: Improved assays and an assay applicable to acrylamide gels. Anal. Biochem..

[44] Beers R.F., Sizer I.W. (1952). A spectrophotometric method for measuring the breakdown of hydrogen peroxide by catalase. J. Biol. Chem..

[45] Fridovich I. (1998). Oxygen toxicity: A radical explanation. J. Exp. Biol..

[46] Chattopadhyay M.K. (2002). Low temperature and oxidative stress. Curr. Sci..

[47] Ramasamy K.P., Mahawar L., Rajasabapathy R., Rajeshwari K., Miceli C., Pucciarelli S. (2023). Comprehensive insights on environmental adaptation strategies in Antarctic bacteria and biotechnological applications of cold adapted molecules. Front. Microbiol..

[48] de Francisco Martínez P., Morgante V., González-Pastor J.E. (2022). Isolation of novel cold-tolerance genes from rhizosphere microorganisms of Antarctic plants by functional metagenomics. Front. Microbiol..

[49] Gocheva Y.G., Krumova E., Slokoska L.S., Gesheva V., Angelova M. (2005). Isolation of filamentous fungi from Antarctica. C. R. Acad. Bulg. Sci..

[50] Gocheva Y.G., Krumova E.T.Z., Slokoska L.S., Miteva J.G., Vassilev S.V., Angelova M.B. (2006). Cell response of Antarctic and temperate strains of *Penicillium* spp. to different growth temperatures. Mycol. Res..

[51] Tosi S., Kostadinova N., Krumova E., Pashova S., Dishliiska V., Spassova B., Vassilev S., Angelova M. (2010). Antioxidant enzyme activity of filamentous fungi isolated from Livingston Island, Maritime Antarctica. Polar Biol..

[52] Kostadinova N., Tosi S., Spassova B., Angelova M. (2017). Comparison of the oxidative stress response of two Antarctic fungi to different growth temperatures. Pol. Polar Res..

[53] Miteva-Staleva J.G. (2017). Low Temperature Stress and Cell Aging in *Antarctic fungi*. Ph.D. Thesis.

[54] Miteva-Staleva J., Krumova E., Spasova B., Angelova M. (2023). Age-related changes in protease activity as cold stress response by *Penicillium* strains from different temperature classes. Pol. Polar Res..

[55] Morita R.Y. (1975). Psychrophilic bacteria. Bacteriol. Rev..

[56] Zucconi L., Pagano S., Fenice M., Selbmann L., Tosi S., Onofri S. (1996). Growth temperature preferences of fungal strains from Victoria Land, Antarctica. Polar Biol..

[57] Onofri S., Selbman L., de Hoog G.S., Grube M., Barreca D., Ruisi S., Zucconi L. (2007). Evolution and adaptation of fungi at boundaries of life. Adv. Space Res..

[58] Rosa L.H., Zani C.L., Cantrell C.L., Duke S.O., Van Dijck P., Desideri A., Rosa C.A., Rosa L.H. (2019). Fungi in Antarctica: Diversity, ecology, effects of climate change and bioprospection for bioactive compounds. Fungi of Antarctica: Diversity, Ecology and Biotechnological Applications.

[59] Varrella S., Barone G., Tangherlini M., Rastelli E., Dell’Anno A., Corinaldesi C. (2021). Diversity, ecological role and biotechnological potential of Antarctic marine fungi. J. Fungi.

[60] Robinson C.H. (2001). Cold adaptation in Arctic and Antarctic fungi. New Phytol..

[61] Martorell M.M., Ruberto L.A.M., Fernández P.M., De Figueroa L.I.C., Mac Cormack W.P. (2018). Biodiversity and enzymes bioprospection of Antarctic filamentous fungi. Antarct. Sci..

[62] Carreiro M.M., Koske R.E. (1992). Room temperature isolations can bias against selection of low temperature microfungi in temperate forest soils. Mycologia.

[63] Ruisi S., Barreca D., Selbmann L., Zucconi L., Onofri S. (2007). Fungi in Antarctica. Rev. Environ. Sci. Biotechnol..

[64] Gomes E.C.Q., Gonçalves V.N., da Costa M.C., de Freitas José Cota G., Santos D.A., Johann S., Oliveira J.B.S., da Paixão T.A., Convey P., Rosa L.H. (2024). Pathogenicity of psychrotolerant strains of Antarctic *Pseudogmynoascus* fungi reveals potential opportunistic profiles. Microbe.

[65] Tsuji M. (2016). Cold-stress responses in the Antarctic basidiomycetous yeast *Mrakia blollopis*. R. Soc. Open Sci..

[66] Fuentes M.E., Quiñones R.A., Gutiérrez M.H., Pantoja S. (2015). Effects of temperature and glucose concentration on the growth and respiration of fungal species isolated from a highly productive coastal upwelling ecosystem. Fungal Ecol..

[67] Wang M., Tian J., Xiang M., Liu X. (2017). Living strategy of cold-adapted fungi with the reference to several representative species. Mycology.

[68] Bateman R.M., Sharpe M.D., Singer M., Ellis C.G. (2017). The effect of sepsis on the erythrocyte. Int. J. Mol. Sci..

[69] Moisa E., Negoita S., Corneci D. (2018). Understanding red blood cell rheology in sepsis and its role in clinical practice. from biomolecular aspects to possible therapeutic interventions. Eur. J. Clin. Res..

[70] Aguirre J., Hansberg W., Navarro R. (2006). Fungal responses to reactive oxygen species. Med. Mycol..

[71] Gessler N.N., Averýanov A.A., Belozerskaya T.A. (2007). Reactive oxygen species in regulation of fungal development. Biochemistry.

[72] da Silva M.S., Gomes V.M., Taveira G.B., de Azevedo dos Santos L., Maracahipes Á.C., Rodrigues R., de Oliveira Carvalho A., Fernandes K.V.S., Oliveira A.E.A. (2021). Bifunctional Inhibitors from Capsicum Chinense Seeds with Antimicrobial Activity and Specific Mechanism of Action Against Phytopathogenic Fungi. Protein Pept. Lett..

[73] Oiki S., Nasuno R., Urayama S.I., Takagi H., Hagiwara D. (2022). Intracellular production of reactive oxygen species and a DAF-FM-related compound in *Aspergillus fumigatus* in response to antifungal agent exposure. Sci. Rep..

[74] Thorpea G.W., Reodicaa M., Daviesb M.J., Heerenc G., Jarolimd S., Pillaya B., Breitenbachd M., Higginsa V.T.J., Dawes I.W. (2013). Superoxide radicals have a protective role during H_2_O_2_ stress. Mol. Biol. Cell.

[75] Papapostolou I., Georgiou C.D. (2010). Superoxide radical is involved in the sclerotial differentiation of filamentous phytopathogenic fungi: Identification of a fungal xanthine oxidase. Fungal Biol..

[76] Krumova E.Z., Pashova S.B., Dolashka-Angelova P.A., Stefanova T., Angelova M.B. (2009). Biomarkers of oxidative stress in the fungal strain *Humicola lutea* under copper exposure. Process Biochem..

[77] Ai D., Wu T., Ge Z., Ying Z., Sun S., Huang D., Zhang J. (2024). The coupling effect promotes superoxide radical production in the microalgal-fungal symbiosis systems: Production, mechanisms and implication for Hg (II) reduction. J. Hazard. Mater..

[78] Liu R., Zhang X., Ren A., Shi D.K., Shi L., Zhu J., Yu H.S., Zhao M.W. (2018). Heat stress-induced reactive oxygen species participate in the regulation of HSP expression, hyphal branching and ganoderic acid biosynthesis in *Ganoderma lucidum*. Microbiol. Res..

[79] Davies K.J., Goldberg A.L. (1987). Oxygen radicals stimulate intracellular proteolysis and lipid peroxidation by independent mechanisms in erythrocytes. J. Biol. Chem..

[80] Neves-da-Rocha J., Santos-Saboya M.J., Lopes M.E.R., Rossi A., Martinez-Rossi N.M. (2023). Insights and perspectives on the role of proteostasis and heat shock proteins in fungal infections. Microorganisms.

[81] Yaakoub H., Mina S., Calenda A., Bouchara J.-P., Papon N. (2022). Oxidative stress response pathways in fungi. Cell. Mol. Life Sci..

[82] Ezraty B., Gennaris A., Barras F., Collet J.F. (2017). Oxidative stress, protein damage and repair in bacteria. Nat. Rev. Microbiol..

[83] González-Tortuero E., Rodríguez-Rojas A. (2023). A hypothesis about the influence of oxidative stress on amino acid protein composition during evolution. Front. Ecol. Evol..

[84] Javidi M.R., Maali-Amiri R., Poormazaheri H., Niaraki M.S., Kariman K. (2022). Cold stress-induced changes in metabolism of carbonyl compounds and membrane fatty acid composition in chickpea. Plant Physiol. Biochem..

[85] Ergin S., Altintas F. (2024). Effects of cold stress on protein metabolism of certain walnut cultivars. Front. Life Sci. Relat. Technol..

[86] Iqbal M.J., Kabeer A., Abbas Z., Siddiqui H.A., Calina D., Sharifi-Rad J., Cho W.C. (2024). Interplay of oxidative stress, cellular communication and signaling pathways in cancer. Cell Commun. Signal..

[87] Ouellet F. (2007). Cold acclimation and freezing tolerance in plants. Encyclopedia of Life Sciences.

[88] Suzuki N., Mittler R. (2006). Reactive oxygen species and temperature stresses: A delicate balance between signaling and destruction. Physiol. Plant..

[89] Belinky P.A., Flikshtein N., Lechenko S., Gepstein S., Dosoretz C.G. (2003). Reactive oxygen species and induction of lignin peroxidase in *Phanerochaete chrysosporium*. Appl. Environ. Microbiol..

[90] Abu Bakar N., Lau B.Y.C., ·Gonzalez-Aravena M., Smykla J., Krzewicka B., Karsani S.A., Alias S.A. (2024). Geographical diversity of proteomic responses to cold stress in the fungal genus *Pseudogymnoascus*. Microb. Ecol..

[91] Li Q., McNeil B., Harvey L.M. (2008). Adaptive response to oxidative stress in the filamentous fungus *Aspergillus niger* B1-D. Free Rad. Biol. Med..

[92] Duncan R.S., Keightley A., Lopez A.A., Hall C.W., Koulen P. (2024). Proteomics Analysis on the Effects of Oxidative Stress and Antioxidants on Proteins Involved in Sterol Transport and Metabolism in Human Telomerase Transcriptase-Overexpressing-Retinal Pigment Epithelium Cells. Int. J. Mol. Sci..

[93] D’Amico S., Collins T., Marx J., Feller G., Gerday C. (2006). Psychrophilic microorganisms: Challenges for life. EMBO Rep..

[94] Kania K., Drożak A., Borkowski A., Działak P., Majcher K., Sawicka P.D., Zienkiewicz M. (2023). Mechanisms of temperature acclimatisation in the psychrotolerant green alga *Coccomyxa subellipsoidea* C-169 (*Trebouxiophyceae*). Physiol. Plant..

[95] Hossain A., Gnanagobal H., Cao T., Chakraborty S., Chukwu-Osazuwa J., Soto-Dávila M., Vasquez I., Santander J. (2024). Role of cold shock proteins B and D in *Aeromonas salmonicida* subsp. *salmonicida* physiology and virulence in lumpfish (*Cyclopterus lumpus*). Infect. Immun..

[96] Sharma P., Jha A.B., Dubey R.S., Pessarakli M. (2012). Reactive Oxygen Species, Oxidative Damage, and Antioxidative Defense Mechanism in Plants Under Stressful Conditions. J. Bot..

[97] Eh T.J., Jiang Y., Jiang M., Li J., Lei P., Ji X., Kim H.I., Zhao X., Meng F. (2024). The role of trehalose metabolism in plant stress tolerance. J. Adv. Res..

[98] Duveau F., Cordier C., Chiron L., Le Bec M., Pouzet S., Séguin J., Llamosi A., Sorre B., Di Meglio J.-M., Hersen P. (2024). Yeast cell responses and survival during periodic osmotic stress are controlled by glucose availability. eLife.

[99] Zhu Q., Wijnants S., Feil R., Van Genechten W., Vergauwen R., Van Goethem O., Lunn J.E., Van Ende M., Van Dijck P. (2025). The stress-protectant molecule trehalose mediates fluconazole tolerance in *Candida glabrata*. Antimicrob. Agents Chemother..

[100] François J.M., Walther T., Parrou J.L., Liu Z.L. (2012). Genetics and regulation of glycogen and trehalose metabolism in *Saccharomyces cerevisiae*. Microbial Stress Tolerance for Biofuels, Microbiology Monographs.

[101] Ribeiro G.D., de Holanda Paranhos L., Eleutherio E.C.A. (2024). Trehalose promotes biological fitness of fungi. Fungal Biol..

[102] Jules M., Guillou V., François J., Parrou J.L. (2004). Two distinct pathways for trehalose assimilation in the yeast Saccharomyces cerevisiae. Appl. Environ. Microbiol..

[103] Da Costa Morato Nery D., Da Silva C.G., Mariani D., Fernandes P.N., Pereira M.D., Panek A.D., Eleutherio E.C.A. (2008). The role of trehalose and its transporter in protection against reactive oxygen species. Biochim. Biophys. Acta.

[104] Jain N.K., Roy I. (2009). Effect of trehalose on protein structure. Protein Sci..

[105] Mejía-Barajas J.A., Montoya-Pérez R., Salgado-Garciglia R., Aguilera-Aguirre L., Cortés-Rojo C., Mejía-Zepeda R., Arellano-Plaza M., Saavedra-Molina A. (2017). Oxidative stress and antioxidant response in a thermotolerant yeast. Braz. J. Microbiol..

[106] Chu X.T., Fu J.J., Sun Y.F., Xu Y.M., Miao Y.J., Xu Y.F., Hu T.M. (2016). Effect of arbuscular mycorrhizal fungi inoculation on cold stress-induced oxidative damage in leaves of *Elymus nutans* Griseb. S. Afr. J. Bot..

[107] Yovchevska L., Miteva-Staleva J., Dishliyska V., Stoyancheva G., Gocheva Y., Abrashev R., Spasova B., Angelova M., Krumova E. (2025). Response to salt stress of the halotolerant filamentous fungus *Penicillium chrysogenum* P13. Molecules.

[108] Pedranzani H.E., Gutiérrez M.H., Arias S.M., Zapico M.G., Ruiz-Lozano J.M. (2021). Arbuscular mycorrhiza interaction with *Medicago sativa* plants: Study of abiotic stress tolerance in sustainable agriculture. AIA.

[109] Priya Reddy Y.N., Oelmüller R. (2024). Lipid peroxidation and stress-induced signalling molecules in systemic resistance mediated by azelaic acid/azelaic acid induced1: Signal initiation and propagation. Physiol. Mol. Biol. Plants.

[110] Wang Y., Li Y., Wang J., Xiang Z., Xi P., Zhao D. (2021). Physiological changes and differential gene expression of tea plants (*Camellia sinensis* (L.) Kuntze var. *niaowangensis* Q.H. Chen) under cold stress. DNA Cell Biol..

[111] Raza A., Charagh S., Najafi-Kakavand S., Abbas S., Shoaib Y., Anwar S., Sharifi S., Lu G., Siddique K.H.M. (2023). Role of phytohormones in regulating cold stress tolerance: Physiological and molecular approaches for developing cold-smart crop plants. Plant Stress.

[112] Mansilla M.C., de Mendoza D., Geiger O. (2016). Regulation of membrane lipid homeostasis in bacteria upon temperature change. Biogenesis of Fatty Acids, Lipids and Membranes. Handbook of Hydrocarbon and Lipid Microbiology.

[113] Tsaturyan V., Poghosyan A., Toczyłowski M., Pepoyan A. (2022). Evaluation of malondialdehyde levels, oxidative stress and host-bacteria interactions: *Escherichia coli* and *Salmonella* Derby. Cells.

[114] Naguib M., Feldman N., Zarodkiewicz P., Shropshire H., Biamis C., El-Halfawy O.M., McCain J., Dezanet C., Décout J.L., Chen Y. (2022). An evolutionary conserved detoxification system for membrane lipid-derived peroxyl radicals in Gram-negative bacteria. PLoS Biol..

[115] Li J., Feng L., Li D., Liu X., Pan Y., He J., Zhang J. (2022). ROS regulate NCF2, key metabolic enzymes and MDA levels to affect the growth of *Fusarium solani*. Agriculture.

[116] González-Guerrero M., Cano C., Azcón-Aguilar C., Ferrol N. (2007). *GintMT1* encodes a functional metallothionein in *Glomus intraradices* that responds to oxidative stress. Mycorrhiza.

[117] Debiane D., Calonne M., Fontaine J., Laruelle F., Grandmougin-Ferjani A., Lounes-Hadj Sahraoui A. (2011). Lipid content disturbance in the arbuscular mycorrhizal, *Glomus irregulare* grown in monoxenic conditions under PAHs pollution. Fungal Biol..

[118] Siliakus M.F., van der Oost J., Kengen S.W.M. (2017). Adaptations of archaeal and bacterial membranes to variations in temperature, pH and pressure. Extremophiles.

[119] Sazanova K.V., Senik S.V., Kirtsideli I.Y., Shavarda A.L. (2019). Metabolomic profiling and lipid composition of arctic and Antarctic strains of Micromycetes *Geomyces pannorum* and *Thelebolus microsporus* grown at different temperatures. Microbiology.

[120] Chen Y., Yang X., Zhang L., Wu Q., Li S., Gou J., He J., Zhang K., Li S., Niu X. (2023). Tryptophan-centered metabolic alterations coincides with lipid-mediated fungal response to cold stress. Heliyon.

[121] Ianutsevich E.A., Danilova O.A., Grum-Grzhimaylo O.A., Tereshina V.M. (2024). Membrane lipids and osmolytes in the response of the acidophilic basidiomycete *Phlebiopsis gigantea* to heat, cold, and osmotic shocks. Int. J. Mol. Sci..

[122] Chattopadhyay M.K., Raghu G., Sharma Y.V.R.K., Biju A.R., Rajasekharan M.V., Shivaji S. (2011). Increase in Oxidative Stress at Low Temperature in an Antarctic Bacterium. Curr. Microbiol..

[123] Liu Y., Zhang N., Ma J., Zhou Y., Wei Q., Tian C., Fang Y., Zhong R., Chen G., Zhang S. (2023). Advances in cold-adapted enzymes derived from microorganisms. Front. Microbiol..

[124] Chovanová K., Böhmer M., Poljovka A., Budiš J., Harichová J., Szemeš T., Zámocký M. (2020). Parallel molecular evolution of catalases and superoxide dismutases-focus on thermophilic fungal genomes. Antioxidants.

[125] Fiedurek J., Gromada A., Słomka A., Korniłowicz-Kowalska T., Kurek E., Melke J. (2003). Catalase activity in arctic microfungi grown at different temperatures. Acta Biol. Hung..

[126] Glanz V.Y., Kashirskikh D.A., Grechko A.V., Yet S.F., Sobenin I.A., Orekhov A.N. (2020). Sialidase activity in human blood serum has a distinct seasonal pattern: A pilot study. Biology.

[127] Yadav J., Verma A.K., Garg R.K., Ahmad K., Mahdi A.A., Srivastava S. (2020). Sialic acid associated with oxidative stress and total antioxidant capacity (TAC) expression level as a predictive indicator in moderate to severe Alzheimer’s disease. Exp. Gerontol..

[128] Abe C., Yi Y., Hane M., Kitajima K., Sato C. (2019). Acute stress-induced change in polysialic acid levels mediated by sialidase in mouse brain. Sci. Rep..

[129] Eneva R., Engibarov S., Abrashev R., Krumova E., Angelova M. (2021). Sialic acids, sialoconjugates and enzymes of their metabolism in fungi. Biotechnol. Biotechnol. Equip..

[130] Ogasawara Y., Namai T., Yoshino F., Lee M.C., Ishii K. (2007). Sialic acid is an essential moiety of mucin as a hydroxyl radical scavenger. FEBS Lett..

[131] Yida Z., Imam M., Ismail M., Ismail N., Ideris A., Abdullah M. (2015). High fat diet-induced inflammation and oxidative stress are attenuated by N-acetylneuraminic acid in rats. J. Biomed. Sci..

[132] Zhu W., Chen X., Yuan L., Wu J., Yao J. (2020). Degradation Kinetics and Shelf Life of *N*-acetylneuraminic Acid at Different pH Values. Molecules.

[133] Pawluczyk I.Z.A., Najafabadi M.G., Brown J.R., Bevington A., Topham P.S. (2015). Sialic acid supplementation ameliorates puromycin aminonucleoside nephrosis in rats. Lab. Investig..

[134] Li D., Lin Q., Luo F., Wang H. (2024). Insights into the Structure, Metabolism, Biological Functions and Molecular Mechanisms of Sialic Acid: A Review. Foods.

